# Supplementary material for: HIV-1-Infected Human Macrophages, by Secreting RANK-L, Contribute to Enhanced Osteoclast Recruitment
Source: Int J Mol Sci. 2020 Apr 30;21(9):3154. doi: 10.3390/ijms21093154 (PMC7246503; doi:10.3390/ijms21093154)
Supplement: Supplementary file 1 [file ijms-21-03154-s001.pdf]

International Journal of
*Molecular Sciences*

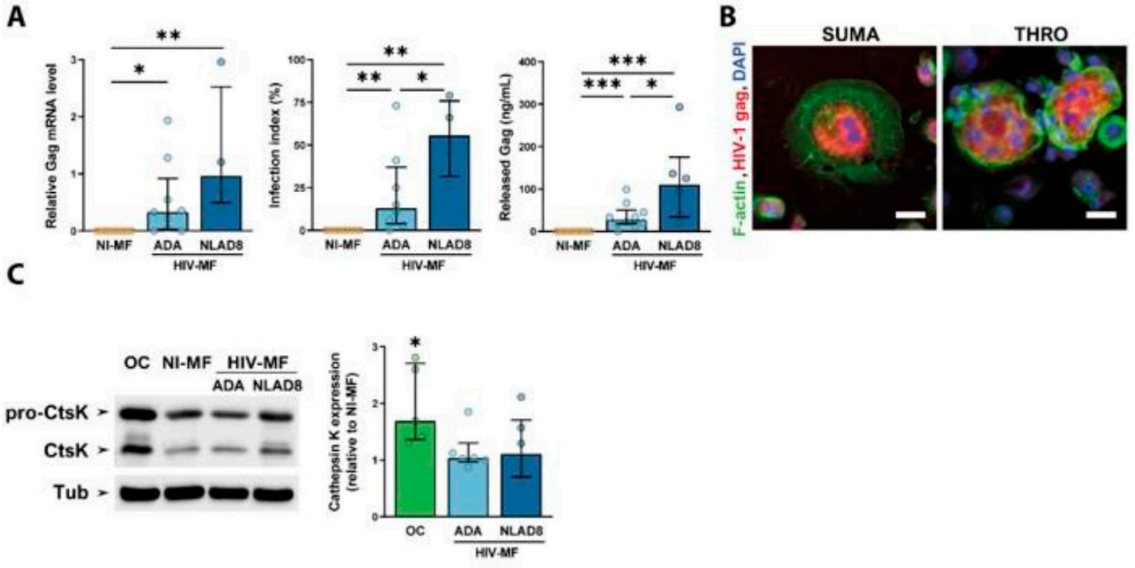


**Figure S1.** (**A**) Infection of MF with HIV-1 (ADA or NLAD8 strain) was evaluated by measuring the expression of the viral gene Gag by RT-qPCR (left panel, results are normalized to GAPDH expression), calculating the fusion index by IF (middle) and measuring p24 release in the supernatant by ELISA (right). $n$ = 4 to 9 donors. (**B**) Representative IF images of MF infected with Transmitted/founder strains SUMA (left) and THRO (right) after staining of HIV-p24 (red), F-actin (green), and nuclei (DAPI, blue). Scale bar, 20 µm. (**C**) Cathepsin K (Ctsk) protein expression level was measured by Western blot in lysates from OC and infected (HIV-MF, ADA or NLAD8 strain) or uninfected MF (NI-MF). Tubulin was used as loading control. A representative blot and quantification of CtsK level relative to autologous NI-MF are shown. Histograms represent median and error bars are interquartile range, $n$ = 6 donors. * $p \leq 0.05$; ** $p \leq 0.001$; *** $p \leq 0.001$, ns: not significantly different.

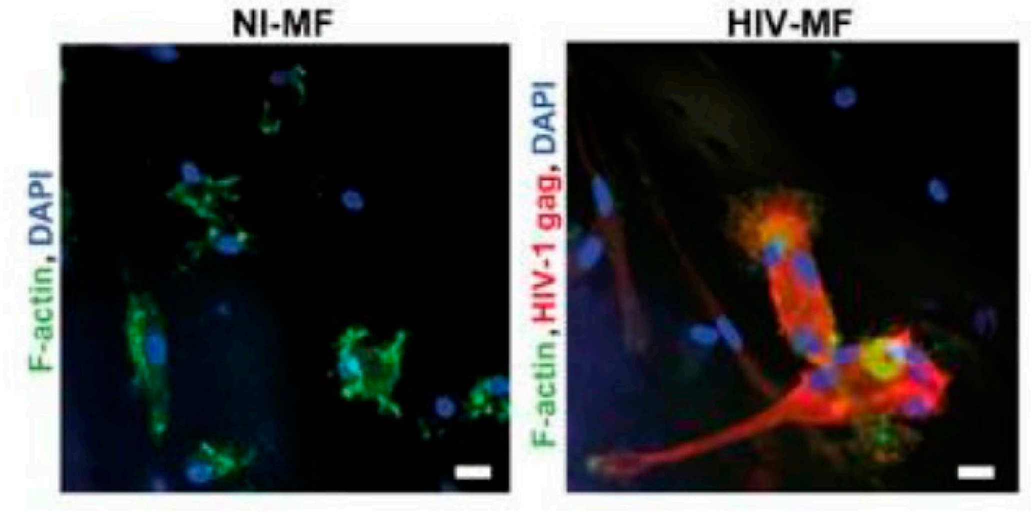


**Figure S2.** Monocytes were seeded on bone slices, differentiated into MF for 7 days. Cells were then infected or not with HIV-1 and all cells were fixed at day 14. Representative IF images of cells after staining for HIV-p24 (red), F-actin (green), and nuclei (DAPI, blue). Scale bar, 10 µm.

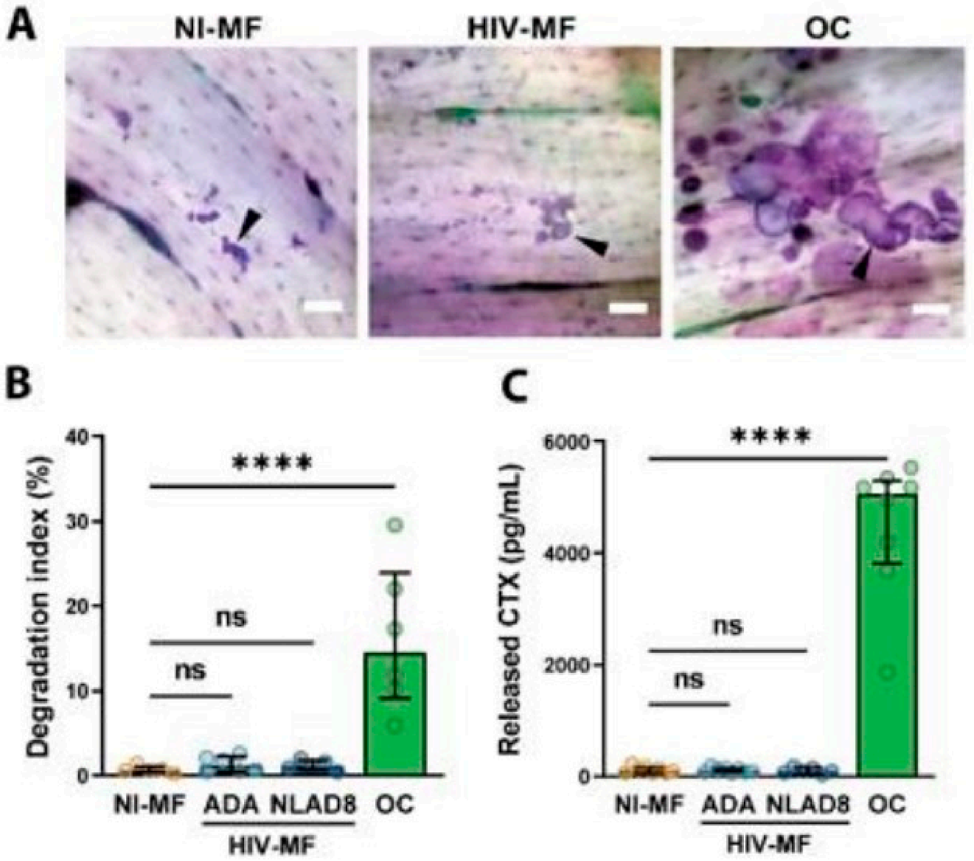


**Figure S3.** HIV-1 infection of MF does not enhance their bone degradation activity. Monocytes were seeded on bone slices and differentiated into MF or OC. At day 7, MF were infected with HIV-1 (HIV-MF) or not (NI-MF). At day 14, the supernatant was collected and cells were removed and bone slices were stained with Toluidine blue. (**A**) Representative images of the surface of bone slices cultured with OC, NI-MF of HIV-MF (NLAD8 strain). Resorbed areas are revealed in purple (arrowheads). Scale bar, 10 µm. (**B**) Quantification of the resorbed area relative to total bone surface. n = 5 to 6 donors. (C) Concentration of the bone degradation marker CTX in the supernatants was measured by ELISA. $n$ = 6 to 8 donors. Histograms represent median and error bars are interquartile range. * $p \leq 0.05$; **** $p \leq 0.0001$, ns: not significantly different.

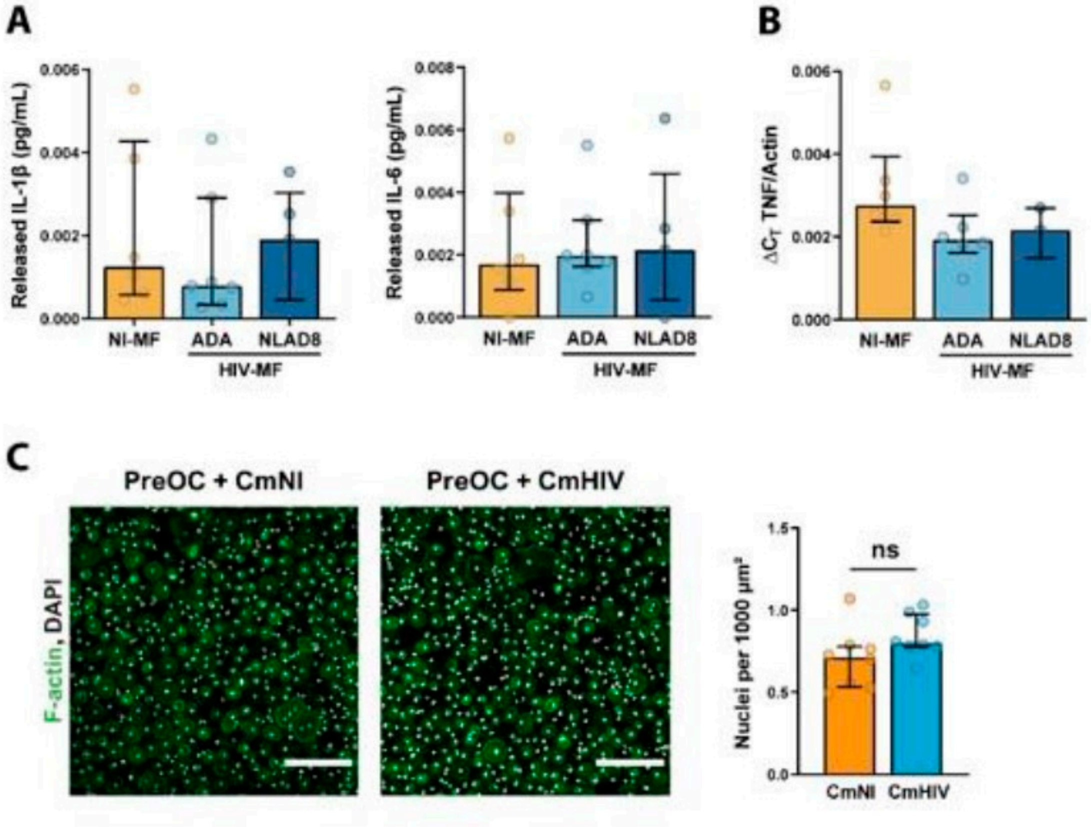


**Figure S4.** (**A**,**B**) MF were infected or not with HIV-1 (ADA or NLAD8 strain) for 10 days and the expression of pro-inflammatory cytokines was evaluated. (**A**) Released IL-1β (left) and IL6 (right) was measured in the supernatants by ELISA. Bars represent median, n = 5 to 7 donors. (**B**) TNF$\alpha$ expression was measured by RT-qPCR (results are normalized to actin). Histograms represent median and error bars are interquartile range, *n* = 3 to 6 donors. (**C**) Monocytes were differentiated for 3 days in presence of sub-optimal concentrations of RANK-L (PreOC) and then exposed to the supernatant of infected (CmHIV) or uninfected (CmNI) MF for additional 10 days. Cells were then fixed and viability was quantified by IF as the number of nuclei per surface area. Scale bar, 200 µm. Histograms represent median and error bars are interquartile range, n = 8 donors. ns: not significantly different.

**Table 1.** List of primers used for cDNA amplification.

| Protein | Gene | Forward Primer (5′-3′) | Reverse Primer (5′-3′) |
|---|---|---|---|
| β-Actin | *ACTB* | TCCCTGGAGAAGAGCTACGA | AGGAAGGAAGGCTGCAAGAG |
| ATP6v1 c1 | *ATP6V1 C1* | CGGCAACTTCAAAGAACAAT | AAGCCCAACAGGAACCACAC TG |
| Catheps in K | *CTSK* | GATGACTGGACTCAAAGTACC | AAGCCCAACAGGAACCACAC TG |
| HIV-1 Gag | *Gag* | AGTGGGGGGACATCAAGCAGCCA TGCAAT | TGCTATGTCACTTTCCCCTTGG TTCTCT |
| NFATc 1 | *NFATC1* | CACCGCATCACAGGGAAGAC | GCACAGTCAATGACGGCTC |
| RhoE | *RND3* | GACACTTCGGGTTCTCCT | CAAAGCAAATCAGCACAGC |
| TNF$\alpha$ | *TNF* | GAGGCCAAGCCCTGGTATG | CGGGCCGATTGATCTCAGC |
| TRAP | *ACP5* | TGACTTCCTCAGCCAGCA | AGCCCACGCCATTCTCATCTT G |

| | | | |
|---|---|---|---|
| β3 integrin | *ITGB3* | CCTGCTCATCTGGAAACTC | TGGGTTGTTGGCTGTGTC |

**Table S2.** List of primary antibodies used and applications.

| Target Protein | Host Specie | Clonality | Supplier | Application | Catalog Number |
|---|---|---|---|---|---|
| Anti-human HRP | Goat | Polyclonal | Sigma | ELISA | A0170 |
| HIV (detection) | Human | Polyclonal | NIH AIDS Reagent Program | ELISA | 3957 |
| HIV-1-gag (Capture) | Mouse | Monoclonal (IgG1 κ), clone 183-H12-5C | NIH AIDS Reagent Program | ELISA | 3537 |
| Anti-mouse AF555 | Goat | Polyclonal | Cell Signaling | IF | 4084 |
| HIV-p24 | Mouse | Monoclonal (IgG1), clone FH190-1-1 | Beckman Coulter | IF | KC57-RD1 |
| Vinculin | Mouse | Monoclonal (IgG1), clone hVIN-1 | Sigma | IF | V9131 |
| Anti-mouse HRP | Goat | Polyclonal | Dako | WB | P0447 |
| Anti-rabbit HRP | Goat | Polyclonal | Dako | WB | P0448 |
| Cathepsin K | Rabbit | Polyclonal | Abcam | WB | ab19027 |
| RhoE | Mouse | Monoclonal (IgG1) | Cell Signaling | WB | 3664 |
| Tubulin | Mouse | Monoclonal (IgG1), clone B5-1-2 | Sigma | WB | T5168 |
| β3 integrin | Rabbit | Polyclonal | Cell Signaling | WB | 4702 |
